# Genetic diversity of ruminant *Pestivirus* strains collected in Northern Ireland between 1999 and 2011 and the role of live ruminant imports

**DOI:** 10.1186/s13620-016-0066-5

**Published:** 2016-06-27

**Authors:** Maria P. Guelbenzu-Gonzalo, Lynsey Cooper, Craig Brown, Sam Leinster, Ronan O’Neill, Liam Doyle, David A. Graham

**Affiliations:** 1 Agri-Food and Biosciences Institute, Veterinary Sciences Division, Belfast, UK; 2 Department of Agriculture, Food and the Marine, Central Veterinary Research Laboratory, Backweston, Ireland; 3 Department of Agriculture, Environment and Rural Affairs of Northern Ireland, Belfast, UK; 4 Animal Health Ireland, Carrick-on-Shannon, Ireland

**Keywords:** Bovine viral diarrhoea virus, Phylogenetic analysis, 5’untranslated region, Importation

## Abstract

**Background:**

The genus pestivirus within the family *Flaviviridae* includes bovine viral diarrhoea virus (BVDV) types 1 and 2, border disease virus (BDV) and classical swine fever virus. The two recognised genotypes of BVDV are divided into subtypes based on phylogenetic analysis, namely a-p for BVDV-1 and a-c for BVDV-2.

**Methods:**

Three studies were conducted to investigate the phylogenetic diversity of pestiviruses present in Northern Ireland. Firstly, pestiviruses in 152 serum samples that had previously tested positive for BVDV between 1999 and 2008 were genotyped with a RT-PCR assay. Secondly, the genetic heterogeneity of pestiviruses from 91 serum samples collected between 2008 and 2011 was investigated by phylogenetic analysis of a 288 base pair portion of the 5’ untranslated region (UTR). Finally, blood samples from 839 bovine and 4,437 ovine animals imported in 2010 and 2011 were tested for pestiviral RNA. Analysis of animal movement data alongside the phylogenetic analysis of the strains was carried out to identify any links between isolates and animal movement.

**Results:**

No BVDV-2 strains were detected. All of the 152 samples in the first study were genotyped as BVDV-1. Phylogenetic analysis indicated that the predominant subtype circulating was BVDV-1a (86 samples out of 91). The remaining five samples clustered close to reference strains in subtype BVDV-1b. Out of the imported animals, 18 bovine samples tested positive and 8 inconclusive (Ct ≥36), while all ovine samples were negative. Eight sequences were obtained and were defined as BVDV-1b. Analysis of movement data between herds failed to find links between herds where BVDV-1b was detected.

**Conclusion:**

Given that only BVDV-1a was detected in samples collected between 1968 and 1999, this study suggests that at least one new subtype has been introduced to Northern Ireland between 1999 and 2011 and highlights the potential for importation of cattle to introduce new strains.

## Background

Two genotypes of bovine viral diarrhoea virus (BVDV) referred to as BVDV types 1 and 2 (BVDV-1, BVDV-2), are currently recognised. BVDV-2 was first detected when virulent strains caused significant losses among cattle in North America in the 1990s [1]. Subsequently, BVDV-2 has been found in many other countries, with the first reported detection in the United Kingdom in 2002 [2]. While BVDV-1 includes at least 18 subtypes (BVDV-1a to 1r) [3–6], BVDV-2 strains are classified into four subtypes (BVDV-2a to 2d) [7–9]. A new species of atypical bovine pestivirus, Hobi virus, (BVDV-3) consisting of viral strains first detected in foetal calf serum (FCS) has also been proposed [10]. The batch of FCS from which Hobi virus was identified was produced in South America and commercialised in European countries [11] and the same viral strain was also subsequently detected in a buffalo from Brazil [12], in a calf in Thailand [13], in calves in Italy [14] and in dairy cattle in India [15].

Other “atypical” pestiviruses of non-bovine origin, including isolates from a giraffe in Kenya, a pronghorn antelope in Africa and Bungowannah virus of pigs in Australia [7, 16, 17] have also been suggested as novel pestiviral species.

The 5’ untranslated region (5’ UTR) of BVDV is highly conserved and has been used as the standard target for diagnostic RT-PCR [18, 19]. The sequence variation of this region is sufficient to enable phylogenetic analysis to assign isolates to genotypes and subtypes [3, 18]. Knowledge of the predominant genotypes in a given region is necessary to ensure that suitable diagnostic tests are used and to assess the protection likely to be afforded by available vaccines, as well as providing surveillance for the emergence of new genotypes or sub-types. Inactivated BVDV vaccines licensed in the United Kingdom and Ireland contain BVDV-1a. It has been estimated than 51 % of dairy herds and 26 % of suckler herds in Northern Ireland vaccinate the adult herd [20]. Vaccines containing BVDV-1 have been shown to provide incomplete protection against type 2 challenge [21–23]. In addition, the ability of vaccines of one BVDV-1 subtype to fully protect against other BVDV-1 subtypes has also been questioned [24, 25]. In 2015, a double deleted modified live vaccine containing two types of a modified live BVDV (BVDV-1 and BVDV-2) has been brought to the market in England, Wales, Northern Ireland and Scotland.

With the exception of a single study comprising 25 sequences [26] and a more recent study confined to the Republic of Ireland [27], there is no published information available on the diversity of ruminant pestiviral strains in Northern Ireland. The current study was undertaken to address this knowledge gap, with emphasis on the genotypes and sub-genotypes present, their relationship to strains present in the rest of the United Kingdom (UK) and in the Republic of Ireland (ROI) and the role of imported cattle and sheep in driving diversity.

## Methods

### Samples and analysis

Three panels of samples were used. All samples were stored at -80 °C.

#### Panel I

One hundred fifty two serum samples submitted from 152 different herds to the Veterinary Sciences Division of the Agri-Food and Biosciences Institute (AFBI-VSD) between 1999 and December 2008 were selected from an archive for determination of viral species by real time RT-PCR. These samples had initially been found positive by a commercial BVDV antigen capture enzyme-linked immunosorbent assay (ELISA) (IDEXX HerdChek BVD Antigen test kit) during routine diagnostic testing.

#### Panel II

As Panel I results indicated a limited diversity, more recent samples were selected to investigate BVDV-1 diversity in greater depth. 91 serum samples submitted from 88 herds to AFBI VSD between 2008 and 2011 were selected for partial genome sequencing. These samples had initially been found positive for BVDV when subjected to routine diagnostic testing by one of the following: commercial BVDV antigen ELISA (as above), virus isolation in foetal calf lung cells or real time RT-PCR assay (Ambion BVDV Taqman AgPath, Life Technologies).

Each sample in Panels I and II was assigned a unique code in the format VSD-YY-XX, with YY being a unique identifier for that year. Available details on the animal from which each sample originated, the reason for submission, and associated clinical signs were collated for Panels I and II.

#### Panel III

Archived RNA samples from 839 bovine and 4,437 ovine EDTA blood samples collected from all ruminants imported into Northern Ireland from both continental Europe and the south eastern (SE) counties of England during 2010 and 2011. Animals were sampled once they reached the receiving herd. These represent a proportion of the blood samples collected from all ruminants (12,285 sheep, 12,167 cattle and 40 from other ruminant species (goats, giraffes, elephants and other camelidae) imported between January 2008 and December 2012 for bluetongue virus (BTV) surveillance (serology and real time RT-PCR).

Retrieved samples were screened for pestiviral RNA by real time RT-PCR, followed by sequencing where appropriate. Each sample was assigned a unique code in the format INT-SCYY-ZZZZ.

### Viral RNA extraction

#### Panels I and II

RNA was extracted from 200 μl of each sample using the RNeasy 96 Universal Tissue 8000 kit (Qiagen, UK) and a Qiagen BioRobot Universal System (Qiagen, UK) according to the manufacturer’s instructions. A volume of 40 μl of RNase-free water was then used to elute the RNA. The extracted RNA was stored at −80 °C until used.

#### Panel III

RNA from Panel II samples was originally extracted for the purpose of BTV surveillance. RNA was extracted using either a manual or an automated protocol. The manual extraction was carried out using a modified QIAmp viral RNA (Qiagen, UK) protocol. Briefly, 40 μl protease was added to 50 μl of the EDTA blood sample. 550 μl of lysis buffer (36:19 ratio of MagNA Pure LC Total Nucleic Acid Isolation Kit and Lysis/Binding Buffer Refill: nuclease free water) was then added, followed by incubation for 15 minutes at 56 °C. RNA was then extracted as per the manufacturer’s instructions with RNA eluted in 50 μl of RNase-free water.

Automated extraction was carried out using a customised robotic protocol (modified QIAamp BioRobot MDX Kit/BioRobot One-for-All kit). Briefly, 40 μl protease was added to 50 μl EDTA blood sample. 550 μl of lysis buffer (as above) was added and sample incubation and subsequent extraction were performed on the Qiagen BioRobot Universal System. RNA was eluted in 70 μl of RNAase free water. Following testing for BTV, RNA was stored at -80 °C.

### Real time RT-PCR testing

#### Panel I

Genotyping was conducted using a commercial real time RT-PCR kit (Cador BVDV Type 1/2 real time RT-PCR Kit, Qiagen, UK) capable of distinguishing between BVDV-1, BVDV-2 and BDV in a triplex reaction. The assay was run on an ABI 7500 Fast instrument (Life Technologies) according to the manufacturer’s instructions except that the reaction volume comprised 20 μl of mastermix and 5 μl RNA.

#### Panel III

Following thawing, 4 μl aliquots of ovine RNA samples were pooled (25 samples per pool). 10.5 μl per pool of ovine RNA was then tested for BVDV RNA with a commercial real time RT-PCR assay (Ambion BVDV Taqman AgPath, Life Technologies) according to manufacturer’s instructions. Samples with a Ct below the reproducible level of detection (RLOD) were considered positive (Ct < 36), those with a 36 ≤ Ct < 45, inconclusive and those with no signal, negative. An extraction control was added to all samples as a positive control for recovery of RNA. Only those samples with a signal for the extraction control and no signal for BVDV were considered negative. Bovine samples were tested without pooling using the same kit. According the manufacturer’s validation report, the assay detects both BVDV-1 and -2 with high sensitivity. Further validation studies carried out in-house (data not shown) indicated that it could also detect a range of BDV strains including S139.

Water extraction negative controls were included at a rate of one to every ten samples. Negative controls containing only the reagents/mastermix (no template controls- NTC) as well as positive controls were included in every run. Only runs with negative results in the water extraction and NTCs and positive results in the positive controls were accepted.

### RT-PCR and sequencing

#### Panel II

The genomic region encoding the highly conserved 5’-UTR of the pestivirus genome was amplified using the QuantiTect kit (Qiagen, UK) with primers 324 (forward: ATG CCC WTA GTA GGA CTA GCA, where W = A or T) and 326 (reverse: TCA ACT CCA TGT GCC ATG TAC) flanking a 288-base pair (bp) fragment as described by Vilcek et al. [18]. The reaction mixture consisted of 12.5 μl of QuantiTect buffer, 0.25 μl of QuantiTect RT enzyme, 0.5 μl of each primer, 6.25 μl of water and 5 μl of template RNA. Cycling conditions were as follows: 50 °C for 30 minutes, 95 °C for 15 minutes, followed by 35 cycles at 94 °C for 1 minute, 60 °C for 1 minute, 72 °C for 1 minute, and a final incubation of 72 °C for 10 minutes. PCR products were separated by gel electrophoresis in 1.5 % agarose gel containing ethidium bromide and examined on a UV transilluminator. DNA bands of the expected sizes were excised from the agarose gel and recovered using QIAquick Purification Kit (Qiagen, UK) according to manufacturer’s protocol. Sequences were then obtained from a commercial provider (Source Bioscience, Ireland).

#### Panel III

Panel III samples were tested with the same primers but different PCR kit and conditions as they were processed in different laboratories. RNA from samples that gave a positive or inconclusive result by real time RT-PCR was subjected to RT-PCR in a reaction mixture consisting of 10 μl of QuantiTect SYBR Green buffer, 0.2 μl of QuantiTect RT enzyme, 2.4 μl of each primer (324 and 326), 3 μl of water and 2 μl of template RNA. Cycling conditions were as follows: 45 °C for 10 minutes, 95 °C for 10 minutes, followed by 40 cycles at 95 °C for 15 seconds, 60 °C for 45 seconds and a final incubation of 72 °C for 10 minutes.

Sequencing of obtained bands was performed using the Big Dye cycle sequencing technology (Applied Biosystems, Life Technologies) and the automated ABI3730 DNA sequencer (Applied Biosystems, Life Technologies).

### Phylogenetic analysis

**Fig. 1:**
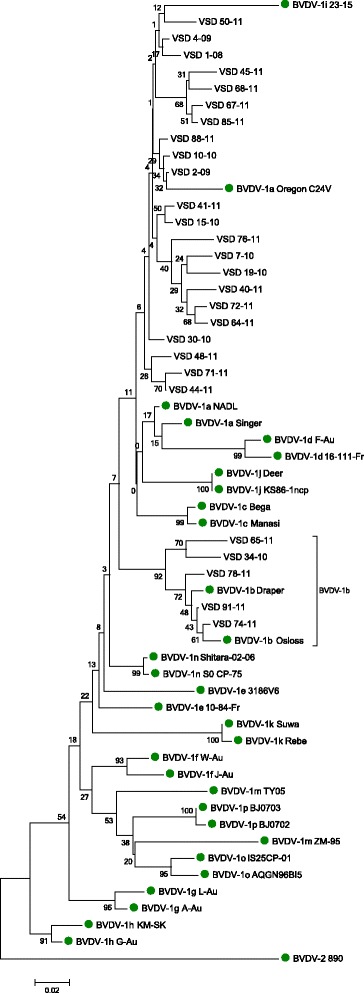
Phylogenetic typing of 27 selected (excluding identical strains) Panel II isolates in the 5’UTR. Reference strains are marked with a circle and the accession numbers for these strains on NCBI Genbank are as follows: BVDV-1a- NADL (M31182), Singer (L32875), Oregon C24V (AB019678); BVDV-1b- Draper (L32880), Osloss (M96687); BVDV-1c- Bega (AF049221), Manasi (EU159702); BVDV-1d- F-Au (AF298065), 16-111-Fr (AF298056); BVDV-1e- 10-84-Fr (AF2980054), 3186 V6 (AF298062); BVDV-1f- J-Au (AF298076), W-Au (AF298073); BVDV-1 g- A-Au (AF298064), L-Au (AF298069); BVDV-1 h- G-Au (AF298066), KM-SK (AF298068); BVDV-1i- 23-15 (AF298059); BVDV-1j- KS86-1-ncp (AB078950), Deer (AB040132); BVDV-1 k- Suwa (AF117699), Rebe (AF299317); BVDV-1 m- ZM-95 (AF526381), TY05 (GU120242); BVDV-1n- Shitara-02-06 (AB359930), So CP-75 (AB359929); BVDV-1o- IS25CP-01 (AB359931), AQGN96B15 (AB300691); BVDV-1p- BJ0702 (GU120248), BJ0703 (GU120249); BVDV-2- 890 (BVU18059). GenBank accession numbers for the 5’ regions sequenced for this paper are KP999051-KP999149

Nucleotide sequences were aligned using MUSCLE software [28]. Phylogenetic trees were calculated using the MEGA programme package version 5 [29] based on the neighbour-joining Kimura two-parameter method [30]. Sequences available from GenBank were also included in aspects of the analysis. These included a series of reference strains (BVDV-1a-k, BVDV-1 m-p, BVDV-2 and BDV; Fig. 1) and strains from cattle in Great Britain (Strong et al. [31]), Northern Ireland and the Republic of Ireland [26] (Fig. 3). The robustness of the phylogenetic trees and the significance of branch orders were determined by bootstrapping method carried out on 1,000 replicates [32]. Nucleotide sequences of the BVDV strains have been submitted to GenBank with accession numbers KP999051-KP999149.

### Animal movement tracing analysis

Herd location (county), supplemented by animal movement data for selected herds, was sourced from the Animal and Public Information System (APHIS) of the Department of Agriculture, Environment and Rural Affairs of Northern Ireland (DAERANI). Five herds from Panel II (Herds A to E) and four herds form Panel III (Herds 1 to 4) plus two associated herds (Assoc 1 and 2) were selected due to the detection of BVDV-1b. For each of these herds a full movement trace of all animals moving into and out of the herd during the period 01/01/2009 to 01/01/2012 was carried out to identify any cattle movements between these herds. This method allowed identification of situations where animals either moved directly from one herd to the other or moved through intermediate herds or markets.

## Results

### Summary of submission details for Panels I and II

#### Panel I

Samples from herds located in all of the six counties of Northern Ireland were included. Of the 125 cases for which a history was submitted, 60 (48 %) reported the animal having diarrhoea, 44 (35.2 %) ill thrift and 22 (17.6 %) oral ulcers. More than one sign per case was provided in some instances. Only a small proportion (7.2 %, 9 cases) made specific mention of BVD or were submitted for herd screening for BVDV. The ages of the animals tested were provided for 75 out of the 152, with 40 % being 1 year of age or less, 52 % were between 1 and 2 years and the remaining 8 % were over 2 years of age.

#### Panel II

All counties in Northern Ireland were again represented, with clinical histories being available for 62 % (56) of the samples. As with Panel I, more than one sign per case was provided in some instances. Of these, 31 (55 %) reported the animal having diarrhoea and 31 (55 %) ill thrift. Relative to Panel I, a much higher proportion of samples for which a history was submitted made specific mention of BVD or were submitted for herd screening for BVDV (30 submissions; 54 %). In 57 % of the submissions (52) the age of the animal was provided. Of these, 56 % (29) were 1 to 2 years old. 38 % (20) were under one year of age and a small number (6 %, 3 submissions) were over two years of age.

### Real time RT-PCR testing

#### Panel I

All samples were found to be BVDV-1 when tested with the Cador BVDV Type 1/2 real time RT-PCR Kit.

#### Panel III

When tested with the TaqMan AgPath real time RT-PCR assay, 18 (2.1 %) of the 839 samples from imported cattle gave a positive result and a further 8 an inconclusive result (0.9 %, Ct ≥36). These 26 samples came from 24 different animals, with two animals each being sampled twice at an interval of 2–3 weeks. Twelve of these animals originated from the Netherlands and twelve from SE England. Some clustering of positive/inconclusive samples was observed according to the date of import, including five animals which were imported on the 21/01/2011, and ten on the 07/04/2011 (Table 1).

**Table 1:** Summary of BVDV positive and inconclusive results obtained when Panel III samples were tested by RT-PCR

Lab Ref No.	Animal Ref	Ct	Result	Sequence obtained	Country of birth	Date of import
INT SC10-648 5	INT 1	36.6	Inc	No	UK	07/09/2010
INT SC10-1219 25	INT 2	37.2	Inc	No	UK	21/01/2011
INT SC10-1220 31	INT 3	33.1	Pos	No	UK	21/01/2011
INT SC10-1221 14	INT 4	38.4	Inc	No	UK	21/01/2011
INT SC10-1267 2	INT 6	33.5	Pos	No	UK	21/01/2011
INT SC10-1195 1	INT 23	34.9	Pos	No	UK	21/01/2011
INT SC11-087 1	INT 18	39.5	Inc	No	UK	22/01/2011
INT SC10-1291 22	INT 8	36.1	Inc	No	UK	08/02/2011
INT SC10-1399 4	INT 7	37.0	Inc	No	NL	23/02/2011
INT SC10-1478 2	INT 9	30.5	Pos	No	NL	11/03/2011
INT SC10-1534 1	INT 11	38.3	Inc	No	UK	19/03/2011
INT SC11-044 4^*^	INT 12	20.3	Pos	Yes	NL	07/04/2011
INT SC11-044 6	INT 13	32.1	Pos	No	NL	07/04/2011
INT SC11-085 2	INT 15	35.3	Pos	No	NL	07/04/2011
INT SC11-085 4^*^	INT 16	32.6	Pos	Yes	NL	07/04/2011
INT SC11-085 10^*^	INT 17	30.9	Pos	Yes	NL	07/04/2011
INT SC11-110 5^*^	INT 19	31.3	Pos	Yes	NL	07/04/2011
INT SC11-110 7^*^	INT 20	30.7	Pos	Yes	NL	07/04/2011
INT SC11-119 2^*^	INT 13	29.6	Pos	Yes	NL	07/04/2011
INT SC11-119 3^*^	INT 12	20.3	Pos	Yes	NL	07/04/2011
INT SC11-119 5	INT 21	32.9	Pos	No	NL	07/04/2011
INT SC11-119 6	INT 22	34.5	Pos	No	NL	07/04/2011
INT SC11-078 1	INT 14	33.9	Pos	No	NL	14/04/2011
INT SC10-1533 1^*^	INT 10	32.4	Pos	Yes	UK	07/04/2011
INT SC11-068 1	INT 24	31.6	Pos	No	UK	18/05/2011
INT SC10-1258 1	INT 5	39.9	Inc	No	UK	11/08/2011

*NL*, Netherlands; *UK*, United Kingdom; ^*^sample sequenced

All 4,437 imported ovine samples gave a negative result.

### Phylogenetic analysis of the 5’UTR region

All positive and inconclusive samples from Panel III were available for further testing. However, PCR products of the expected size suitable for subsequent nucleotide sequence determination were only obtained from eight of the 18 positive samples only (Table 1).

**Fig. 2:**
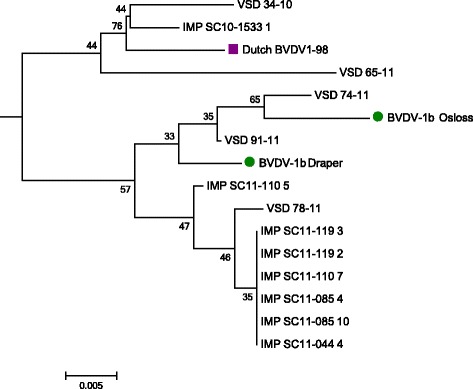
Phylogenetic subtree with all BVDV-1b detected strains in Panels II and III and a Dutch BVDV-1b strain. Reference strains are marked with a circle. Accession numbers for these as per Fig. 1 and Dutch BVDV-1b-98 (AF098156)

**Fig. 3:**
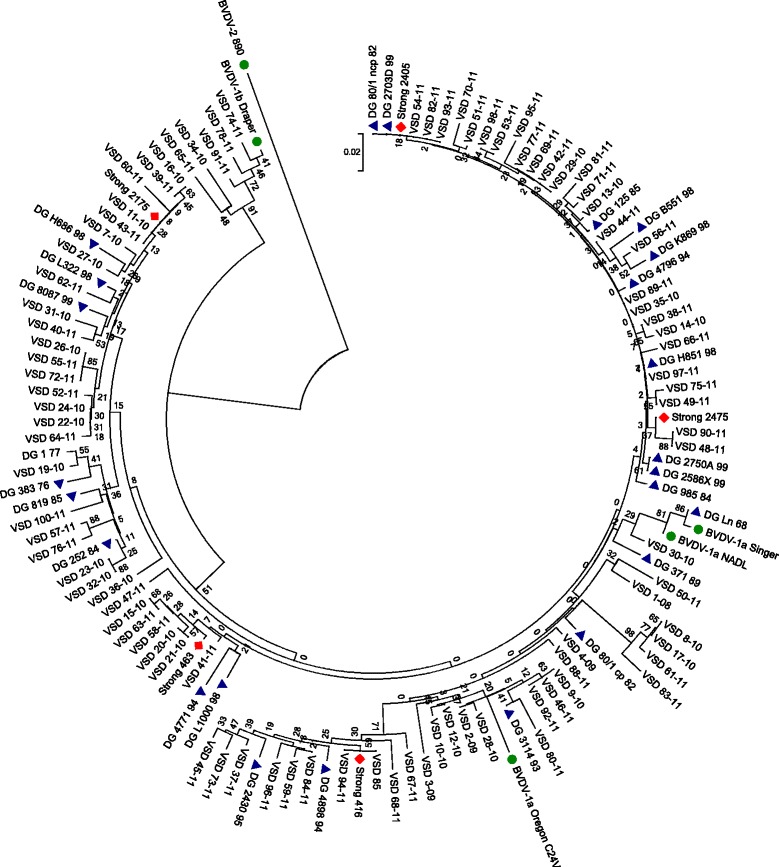
Phylogenetic circle showing the relationship between the 91 samples in Panel II and previous Northern Ireland (NI), Republic of Ireland (ROI) and Great Britain (GB) strains. Relevant reference strains are marked with a dot, previously reported strains from NI and ROI marked with a triangle and previously reported GB strains are marked with a diamond. The accession numbers for these strains on NCBI Genbank are as follows: DG H851 98 (AJ312908) (ROI), DG 125 85 (AJ312909) (NI), DG 4796 94 (AJ312910) (NI), DG B551 98 (AJ312911) (ROI), DG K869 98 (AJ312912) (ROI), DG 2703D 99 (AJ312913) (NI), DG 80/1 ncp 82 (AJ312914) (NI), DG 2586X 99 (AJ312915) (NI), DG 2750A 99 (AJ312916) (NI), DG 985 84 (AJ312917) (NI), DG 371 89 (AJ312918 (NI)), DG L1000 98 (AJ312919) (ROI), DG 4771 94 (AJ312920) (NI), DG 8087 99 (AJ312921) (NI), DG L322 98 (AJ312922) (ROI), DG H686 98 (AJ312923) (ROI), DG 819 85 (AJ312924) (NI), DG 252 84 (AJ312925) (NI), DG 383 76 (AJ312926) (NI), DG 1 77 (AJ312927) (NI), DG Ln 68 (AJ312928) (NI), DG 80/1 cp 82 (AJ312929) (NI), DG 2430 95 (AJ312930) (NI), DG 4898 94 (AJ312931) (NI), DG 3114 93 (AJ312932) (NI), Strong 416 (JQ920052), Strong 463 (JQ920074), Strong 2175 (JQ920096), Strong 2405 (JQ920208), Strong 2475 (JQ920250)

5’UTR sequence data from these 8 Panel III samples and the 91 Panel II samples were aligned with BVDV-1, BVDV-2 and BDV reference strains as well as other UK and Irish strains and phylogenetic analysis was performed (Figs. 1, 2 and 3). The analysis indicated that the predominant subtype circulating in Northern Ireland between 1999 and 2011 was BVDV-1a (86 samples out of 91). Five out of the 91 samples (Panel II) clustered close to reference strains in subtype BVDV-1b, with one of these being detected in 2010 and the remaining four in 2011 (Fig. 2). Two of these originated from herds in county Tyrone, and the other three from counties Armagh, Down and Londonderry. Three of these were suckler herds and two were beef fattening herds, with all having introduced animals within the previous two years.

In contrast to the Panel II sequences, all eight Panel III sequences clustered to reference strains in subtype BVDV-1b (Fig. 2). These eight sequences were obtained from seven animals imported to four herds, two in county Tyrone and two in Antrim. One of the animals (INT 12) was sampled twice, three weeks apart. All seven animals originated from different herds in the Netherlands, although one was imported as a foetus at five months of gestation in a pregnant dam, being subsequently tested as a 2 months old calf (sample INT SC10-1533 1, animal INT 10). All of the Dutch animals including the dam pregnant with INT 10 were imported on the same date (Table 1).

Sequence data from some of the strains shared 100 % identity with sequences generated in previous studies. For example, VSD 84-11, VSD 85-11 and VSD 94-11 showed 100 % identity to UK strain 416 (Fig. 3). VSD 13-10, VSD 53-11, VSD 93-11 and VSD 98-11 showed 100 % identity to UK strain 2405 and VSD 12-10, VSD 43-11 and VSD 88-11 showed 100 % identity to UK strains 467, 2175 and 2521 respectively [31]. Six strains (VSD 35-10, VSD 42-11, VSD 69-11, VSD 77-11, VSD 89-11 and VSD 97-11) showed 100 % identity to Northern Irish strain H851 98 [26].

Although VSD 36-10 seemed close in the tree to BVDV-1i 23-15 (Fig. 1), a blast search showed 98 % identity to UK 2183 (from 2008) and 2634 (from 2009), both BVDV-1a strains.

Six of the eight sequences from the seven imported animals were identical (Fig. 2). Two of these identical sequences belonged to the same animal which was sampled twice and when tested obtained low Ct values on both occasions, consistent with it being BVD persistently infected (Table 1). The remaining four identical sequences originated from animals that yielded higher Ct values. Interestingly, the sequence originated in animal INT 10 (INT SC10-1533 1), born in the UK to a cow recently imported from the Netherlands, related more closely to local strains than to the six identical sequences from imported animals. However, the INT 10 sequence and the local BVDV-1b strains clustered with an earlier BVDV-1b Dutch sequence isolated in 1998, raising the possibility that the local strains reflected an earlier introduction from this source.

### Animal movement tracing analysis

Analysis of movement data for the period 01/01/2009 to 01/01/2012 showed that there were animal movements, mainly through markets, between some Panel II and III herds as well as between Panel II herds (Table 2), but failed to identify a direct linkage between these herds in time and space which would have explained the infection events observed in Panel II herds. In addition, the phylogeny does not support movement as means of spread since the strains don’t cluster with the detected movements (Table 2, Fig. 2).

**Table 2:** Summary of movements detected between selected Panel II and Panel III herds

Herd from which a move occurred	Herd to which a move occurred	Date of Move	Number of animals moved	Description of move type
Herd B (VSD 65-11)	Herd D (VSD 78-11)	27/09/2011	3	Three animals born in February and March 2011 moved through a market from Herd B to Herd D
Herd 3 (INT SC11-110 5 and INT SC11-110 7)	Herd C (VSD 74-11)	21/07/2010	1	One animal born 27/06/2010 moved through a market from Herd 3 to Herd C.
Herd 3 (INT SC11-110 5 and INT SC11-110 7)	Herd C (VSD 74-11)	18/06/2008	1	One animal born 22/05/2008 moved from Herd 3 to Herd C on the 18/06/2008 and was picked up as an animal which moved out of Herd C on the 16/07/2010

## Discussion

In the present study the molecular typing of pestiviral strains from cattle in Northern Ireland, including those imported from other parts of the UK and continental Europe, was performed and the results analysed. This study represents the largest survey of this type undertaken to date in Northern Ireland. The initial phase of the work, using Panel I samples, investigated diversity to the species level. No evidence was found of the presence of BVDV-2 or BDV in Panel I samples. This is in contrast to the situation elsewhere in the UK, where the presence of BVDV-2 and BDV has been reported in cattle, albeit infrequently [2, 33]. The lack of detection of BVDV-2 is encouraging, and taken together with the failure to detect BVDV-2 in two studies in ROI (R O’Neill, personal communication, [27]), supports the hypothesis that the island of Ireland remains free of BVDV-2.

Sequencing of Panel II strains collected between 2008 and 2011, provided a more in-depth analysis of genetic diversity, allowing characterisation to subtype level. A high degree of homogeneity was detected, with BVDV-1a being the predominant subgenotype (86/91 samples; 94.5 %). The remaining 5 strains clustered to BVDV-1b reference strains. This is the first report of BVDV-1b in cattle in Northern Ireland.

Both of the inactivated vaccines currently licenced for use in the UK contain BVDV-1a. These findings indicate that they are well suited, in terms of antigenicity, to provide protection against the predominant challenge strains. Some studies suggest that protection against heterologous BVDV-1 subtypes is inferior to homologous BVDV-1 subtypes [23–25]. Because of this concern, it was important to determine the circulating pestiviral strains. A recently licenced modified live vaccine includes both BVDV-1 and BVDV-2 components. The BVDV-1 strain included (field isolate KE9) is genetically related to several other European virus strains.

The first survey to determine the strain type of pestiviruses in Ireland was carried out in 2001 [26], using 25 field strains collected between 1968 and 1999. All were found to be BVDV-1a. Similar findings were reported in England and Wales in a contemporaneous study [34]. However, more recent studies have shown an increased phylogenetic diversity in England and Wales [31]. Although BVDV-1a was still the predominant subtype detected (77.2 %), an increased frequency of detection of BVDV-1b was found, along with three additional circulating subtypes, BVDV-1e, BVDV-1f and BVDV-Ii. Booth et al. [35] reported similar findings in a recent study of 104 samples from GB. The predominant sub-genotype found was again BVDV-1a (88/104 samples; 85 %). However BVDV-1b, 1e and 1i were also identified and the first BVDV -1d in the UK was reported.

In the ROI a more diverse antigenic variability has also been recently reported [27]. BVDV-1a was still the predominant subgenotype, accounting for 97.5 % (*n* = 317) of the viral isolates. Other subgenotypes detected were BVDV-1b (*n* = 6), BVDV-1d (*n* = 1) BVDV-1e (*n* = 1).

The predominance of BVDV-1a in the UK and Ireland [26, 31, 35] is in contrast to other European countries including Poland [36], the Netherlands [37], Portugal [38], Italy [39] and Spain [40] where BVDV-1b is the most prevalent subtype.

Panel III sera were tested to examine the hypothesis that importation of infected ruminants was driving the detected increase in genetic diversity. Overall, the findings support this hypothesis with 2.1 % (18/839) of samples from imported cattle giving a positive result and a further 8 (0.95 %), an inconclusive result. It was not possible to establish in all cases whether the positive and inconclusive samples were from persistently or transiently infected animals. However, two animals (INT 12 and INT 13, Table 1) tested virus positive on two separate occasions which is consistent with them being PI.

As the BVDV-1b strains in Panel II samples were detected throughout or after the period during which the Panel III BVDV-1b-positive animals were introduced, the possibility of a link between those herds by cattle movement was explored. Observing the clustering in Fig. 2, it was logical to investigate a relation between Herd D (VSD-78-11) and Herds 1, 2 and 3. Although all the herds in Panel II where BVDV-1b was detected had each added animals within the previous 2 years, analysis of movement data failed to show links between the Panel III and Panel II herds that would explain the BVDV-1b strains detected in this study. Nevertheless, a combination between phylogenetic and movement analysis may be used as an epidemiological tool to determine the origin of infection.

None of the pools from the 4,437 samples from sheep imported between September 2010 and November 2011 were positive for the presence of pestivirus RNA. This could reflect a lower risk of introduction of pestiviruses from sheep or a lack of sensitivity of the method used (including the impact of pooling). Our in house validation records for the RT-PCR assay used showed that the analytical sensitivity for the detection of several BDV species subtypes was lower than for the detection of BVDV-1 and BVDV-2 (data not shown). The rise in detection of different BVD strains, including atypical strains [11–13] and the increase in reports of cattle affected by BDV [33, 41] highlights the need to monitor circulating strains and to review accordingly the suitability of the diagnostic methods used.

Molecular epidemiology has been used to trace sources and routes of BVDV infections within the Swedish BVD eradication programme [42] and to link animal movements with viral isolates in the UK [35]. The sequence information collated in this study may provide a useful tool to trace the origin of new outbreaks. Since the level of movement of animals between Northern Ireland and other regions of the UK and Ireland is very high, a common database where all the sequence data could be stored would be a useful tool for this purpose.

This study suggests that in terms of BVDV the island of Ireland retains a distinct pattern of strain diversity from continental Europe. The reasons behind this are unclear, but some possibilities include the fact that the vast majority of transboundary cattle movements are outward, the existence of strict importation regulations prior to 1992 the system of cattle production is largely grass-based and the obvious absence of a land-link with other cattle populations.

## Conclusion

In conclusion, this study found strains of BVDV in the cattle population in Northern Ireland to be predominantly BVDV-1a. No evidence of BVDV-2 was found, but the emergence of BVDV-1b in recent years was detected. Testing of samples from imported cattle highlighted this as a route for introduction of new strains leading to increasing viral heterogeneity, emphasising the need to address imports in the context of current eradication programmes in Northern Ireland and the Republic of Ireland.

## Abbreviations

AFBI, Agri-Food and Biosciences Institute; APHIS, Animal and Public Information System; BDV, Border disease virus; BTV, Bluetongue virus; BVDV, Bovine viral diarrhoea virus; DAERANI, Department of Agriculture, Environment and Rural Affairs of Northern Ireland; DAFM, Department of Agriculture, Food and Marine; ELISA, Enzyme-linked immunosorbent assay; FCS, Foetal calf serum; NI, Northern Ireland; NTC, No template control; PI, Persistently infected; RLOD, Reproducible level of detection; ROI, Republic of Ireland; SE, South eastern; UK, United Kingdom; UTR, Untranslated region; VSD, Veterinary Sciences Division.
